# The therapeutic effects of low-intensity pulsed ultrasound in musculoskeletal soft tissue injuries: Focusing on the molecular mechanism

**DOI:** 10.3389/fbioe.2022.1080430

**Published:** 2022-12-16

**Authors:** Haocheng Qin, Liang Du, Zhiwen Luo, Zhong He, Qing Wang, Shiyi Chen, Yu-Lian Zhu

**Affiliations:** ^1^ Department of Rehabilitation Medicine, Huashan Hospital, Fudan University, Shanghai, China; ^2^ Department of Sports Medicine, Huashan Hospital, Fudan University, Shanghai, China; ^3^ Department of Orthopedics, Kunshan Hospital of Chinese Medicine, Suzhou, China

**Keywords:** low-intensity pulsed ultrasound, mechanism, musculoskeletal injuries, regeneration, inflammation

## Abstract

Musculoskeletal soft tissue injuries are very common and usually occur during both sporting and everyday activities. The intervention of adjuvant therapies to promote tissue regeneration is of great importance to improving people’s quality of life and extending their productive lives. Though many studies have focused on the positive results and effectiveness of the LIPUS on soft tissue, the molecular mechanisms standing behind LIPUS effects are much less explored and reported, especially the intracellular signaling pathways. We incorporated all research on LIPUS in soft tissue diseases since 2005 and summarized studies that uncovered the intracellular molecular mechanism. This review will also provide the latest evidence-based research progress in this field and suggest research directions for future experiments.

## Introduction

Musculoskeletal injuries are very common and usually occur during both sport and everyday activities (Abrams et al., 2012). Musculoskeletal system injuries are also commonly encountered in the hospital, such as inevitable muscle and tendon damage from a surgical approach (Çölbe et al., 2021). Musculoskeletal soft tissues include tendon, ligament, cartilage, joint capsule, and muscle. Except for some serious injuries that require immediate medical attention, a large number of people with more minor soft tissue injuries will remain undiagnosed and untreated because they tend to believe it will not affect their daily lives continuously (Gwyer et al., 2019). As a result, some patients may lose the window for treatment and thereby suffer from a chronic disease. Whether the damage is severe or entering a chronic phase, it unquestionably makes treatment more challenging and lowers patients’ quality of life. In addition, there are certain difficulties in musculoskeletal soft tissue regeneration, such as excessive inflammation, infection, and size defects (Ryan, 2007; Miron et al., 2015; Chen et al., 2021). When the damage exceeds the intrinsic regenerative capabilities, autologous or allogeneic transplantation will be the best choice. However, patients may suffer from donor site complications, rejection, and infection when applying autografts and allografts (Ducic et al., 2020). Therefore, the intervention of adjuvant therapy to promote tissue regeneration is of great importance to improving people’s quality of life and extending their productive lives.

Low-intensity pulsed ultrasound (LIPUS), a form of mechanical stimulation, has become a popular non-invasive therapeutic modality in the fields of traumatology and regenerative medicine (Mei and Zhang, 2021). In past decades, the application of ultrasound in medicine, especially for diagnostics, surgery, and therapy, has been wildly investigated. As reported in both animal and clinical studies, LIPUS can accelerate the healing of fresh fractures, nonunion fractures, and delayed union fractures (Harrison et al., 2016). The effectiveness of LIPUS for ameliorating soft-tissue regeneration and inhibiting inflammatory responses has also been studied experimentally. Recently, increasing research has shown that LIPUS is a promising modality for modulating neural activity (Rabut et al., 2020). The application of LIPUS to clinically accelerate the repair process in humans was first reported by Xavier and Duarte in 1983. In 1994, the U.S. Food and Drug Administration approved LIPUS for clinical use in treating fresh fractures (Harrison et al., 2016). Since then, studies investigating LIPUS therapy to accelerate fracture healing have attracted great attention. Therefore, in the fracture regeneration field, studies on the mechanism of LIPUS are more mature compared to the soft tissue regeneration field. Moreover, considerable literature has summarized the signaling pathways affected by LIPUS in promoting fracture repair. The research on LIPUS in promoting soft tissue repair started relatively late and was less comprehensive. Though many studies focused on the positive results and effectiveness of the LIPUS on soft tissue, the real mechanisms standing behind the LIPUS were much less explored and reported, especially the intracellular mechanistic pathways. In recent years, studies on the influence of ultrasound on intracellular signaling pathways have gradually revealed the deeper biological role of this physical factor. Therefore, in this review, we incorporated all research on LIPUS in soft tissue diseases since 2005 and systematically summarized the pathways affected by ultrasound in tendon, ligament, bone–tendon junction, muscle, articular cartilage, and joint capsule. This review will also provide the latest evidence-based research progress in this field and suggest research directions for future experiments.

## An overview of LIPUS

### Parameters

LIPUS uses sound energy as the output form and produces mechanical vibrations in the targeted deep tissues and cells. The algorithm for the output is low-intensity radiation with pulsed-wave form. The pulse wave consists of cycles of ON and OFF phases. The duty cycle is determined by the different proportions of the ON phases in each cycle, including 20%, 50%, 80%, and 100%. The intensity of a LIPUS is determined by the amplitude during the ON period, which can range from 0.02 to 1 W/cm^2^ spatial and temporal average at frequencies ranging from 1 to 3 MHz. Clinically, the lower the frequency of stimulation, the deeper the tissue is affected. Generally, the thermal effect produced by LIPUS can be ignored with only a 0.5°C fluctuation in temperature after 10 min of exposure. Studies also investigated the most optimal parameters for a specific condition. Salgarella et al. found that at 3 MHz and 1 W/cm^2^ intensity, it maximized C2C12 cell proliferation, while 1 MHz stimulation at 500 mW/cm^2^ intensity maximized C2C12 cell differentiation.

### Biomechanics of LIPUS

In contrast to focused ultrasound, which is clinically used for ablation of breast, prostate, or uterine fibroids (Tempany et al., 2011; Quadri et al., 2018), LIPUS utilizes a short duty cycle and low intensity to minimize temperature elevations, enabling the mechanical effects of acoustic radiation force (pressure) and cavitations to predominate (Harrison et al., 2016).

The acoustic energy generated by ultrasound is produced by a transducer, which emits high-frequency acoustic mechanical waves. The waves transmit through the skin tissues and subsequently create cavitation, acoustic streaming, and mechanical stimulation, which then transform into a series of molecular vibrations including microbubbles, microjets, and collisions around the aimed lesions (Jiang et al., 2019). Cavitation is the main mechanism for causing adjustments to biological tissues, especially through its effect on increased membrane permeability (Wu and Nyborg, 2008). Stable cavitation and transitory cavitation are two different forms of cavitation. The former creates bubbles, and the bubble’s radius fluctuates around an equilibrium value. The latter creates bubbles that fluctuate unsteadily (Feril and Kondo, 2004). This micro-force around targeted tissues and cells could elicit a range of desirable and reproducible bioeffects, which include the deformation of the cell membrane, activation of voltage-gated ion channels, and other biochemical reactions at the cellular level (Krasovitski et al., 2011; Castellanos et al., 2016).

However, in various diseases, the specific intracellular molecular mechanisms between the attempted biological effects of mechanical stress applied to cells and the beneficial therapeutic effects of ultrasound are still being extensively studied.

### Biological mechanism of LIPUS

For now, the biological effects brought about by LIPUS have been harnessed for a range of medical applications including blood–brain barrier disruption for the delivery of neurotherapeutics, neuromodulation, tumor immunomodulation, hemodynamic alterations, and regenerative medicine applications. With the increasing attention to LIPUS therapy, substantial reports are being made to clarify the therapeutic mechanisms of LIPUS *via* its biological effects. The therapeutic effect of the ultrasonic signal also includes its heating effect on the target tissue. Ultrasonic energy is absorbed at a rate corresponding to the density of the tissue as ultrasonic vibrations travel through the body. The temperature of the body tissue rises as a result of the ultrasonic signal being absorbed (Liu et al., 2010). For low-intensity ultrasonic waves, this heating effect is extremely small. However, some enzymes, such as matrix metalloproteinase-1 (MMP-1) and collagenase, are extremely sensitive to temperature changes (Khanna et al., 2009). Therefore, ultrasound therapy may adjust the changes in the biological microenvironment of local tissues through this effect, which always has profound implications. In addition, the pressure gradient produced by the ultrasound can also influence the propagation of extracellular matter to some extent, subsequently causing a series of biological reactions. The vibratory effect of ultrasonic energy on the cell surface also stimulates some mechanically sensitive membrane surface receptors, such as integrin (Xiao et al., 2017) and caveolin (Zheng et al., 2019), resulting in a series of intracellular cascade reactions. In addition, the synaptic excitability of glial cells is also affected in response to LIPUS (Kovacs et al., 2017). The conformation state of channel proteins of ion channels can also be modulated by LIPUS, leading to intracellular alternations of ions. Numerous different ion channels are also mechanosensitive, including voltage-gated Na+ and Ca2+ channels (Tyler et al., 2008), Prieto-1 channels (Prieto et al., 2018), Nav1.5 channels, and K+ channels (Kubanek et al., 2016). The changes in membrane capacitance can also result in the excitation of cells (Plaksin et al., 2017). LIPUS has been proven to initiate capacitive currents in the pure lipid membrane model, but it is yet to be clearly elucidated that it was caused by cavitation (Prieto et al., 2013).

In this review, we mainly discuss and summarize the effects of ultrasound therapy on cell surface receptors and downstream intracellular pathways.

## Mechanisms of LIPUS in different soft tissues

### Tendon, ligament, and bone–tendon junction

LIPUS therapy can be applied to many tendon injuries. Around joints, the attachment of tendons and bones creates the bone–tendon junction (BTJ). However, as a transitional area, its recovery is slower and more challenging than tendon-to-tendon or bone-to-bone healing. In sports, exercise, or auto accidents, injuries involving the BTJ surrounding the knee joint are frequent (Lu et al., 2016). A recent study by Li et al. found that at postoperative weeks 2 and 4, the failure load and stiffness of the supraspinatus tendon–humerus junction were considerably improved in the LIPUS group, which was further confirmed to be associated with macrophages. Chen et al. used a combination of LIPUS and adipose-derived mesenchymal stem cell implantation to treat the rabbit model of partial patellectomy. The result indicated that bone formation was increased and regenerated fibrocartilage was enhanced *via* LIPUS treatment. In the study by Lu et al., LIPUS was detected to be effective in recovering the mechanical properties of the BTJ of the patellar through anti-inflammatory effects (Lu et al., 2016).

Tendinopathy is prevalent and usually shifts into a chronic disease that afflicts the patient’s daily life (Smallcomb et al., 2022). Physical modality therapy, such as extracorporeal shock wave, LIPUS, and low-energy laser therapy, was wildly used as adjunctive strategies for tendinopathy recovery. However, the effectiveness of LIPUS in tendinopathy was still inconclusive. The clinical trials from Warden et al. (2008) and D'Vaz et al. (2006) failed to demonstrate any improvement after LIPUS treatment in patients suffering from patellar tendinopathy and common extensor–supinator tendinopathy. Similarly, the data from Desmeules et al. (2015) revealed that LIPUS did not provide any benefit in adults suffering from rotator cuff tendinopathy. However, Özmen et al. found amelioration in patients with epicondylitis after LIPUS treatment, and LIPUS significantly reduced pain and improved functionality (Özmen et al., 2021). Epicondylitis is known as a common musculotendinous degenerative disorder. Although few relevant clinical research studies reported the meaningful outcomes of LIPUS on tendon injuries, the animal models presented us with a promising future for this technique. Yeung et al. (2006) created partial tenotomy of the Achilles tendon with the treatment of LIPUS and found that LIPUS had a stimulatory effect on tissue regeneration in a ruptured Achilles tendon, with more regular, denser, and better-aligned collagen fibers and higher tensile strength. The data from Jeremias Júnior et al. (2011) also found that LIPUS improved the tensile strength on the 28th postoperative day. When it comes to the mechanism behind these effects, only the studies from Fu et al. (2010) and Kasaka et al. found the upregulation of collagen synthesis after LIPUS. Furthermore, according to Fu et al., LIPUS-mediated decorin and biglycan, which are important for the fibrillogenesis of collagen fibers and their transitory upregulation aiding in the formation of collagen fibrils (Zhang et al., 2009; Reese et al., 2013), were significantly improved. Kasaka et al. found that in the acute phase of inflammation, both COX-2 and prostaglandin E2 (PGE2) receptor 4 expressions were markedly induced in the LIPUS-treated group, and TGF-β1 expression was also improved. A rise in TGF-β1 expression was reported to upregulate fibroblast chemotaxis, fibroblast production, and granulation (Xiao et al., 2012). Nevertheless, it was still unclear which signaling pathway accounted for the upregulation of collagen after LIPUS intervention.

Few studies have investigated the therapeutic effects of LIPUS on ligament regeneration. Warden et al. reported that no significant differences in mechanical properties were found between treated and control ligaments (Warden et al., 2006). Leung et al. found that LIPUS enhanced ligament healing by upregulating the expression of TGF-β1 in a rat model of medial collateral ligament transection (Leung et al., 2006). Still, no involved intracellular pathway was discovered.

From the previous research on LIPUS’s therapeutic effects on the injury model of tendon, ligament, and bone–tendon junction, we found that studies were limited to phenotypes of LIPUS treatment effects. Therefore, more *in vitro* and *in vivo* experiments mimicking these three clinical diseases should be put forward to validate the underlying mechanism. In addition, the review from Millar et al. enlightened us that we could utilize the mechanisms/pathophysiology of tendinopathy as a starting point, such as extracellular matrix dysregulation, oxidative injury, apoptotic pathways, and resolution pathways, to investigate the LIPUS-mediated involved pathway (Millar et al., 2021). It is also worthwhile to investigate the clinical curative effect of LIPUS when combined with other therapies in these three models.

### Skeletal muscle

Skeletal muscle injuries are one of the most common lesions occurring in sports and daily activities. A mild lesion can be completely recovered from, but a severe lesion prevents muscle fibers from regaining their pre-injury states, which will exert negative impacts on the quality of daily life. Following an injury, muscle tissue heals through continuous processes that restore the tissue’s structures and functionality. The initial phase, known as the inflammatory phase, is marked by the development of hematomas and inflammatory responses. The next stage is the repair phase, which includes the activation of satellite cells, clearance of the necrotic tissue, and myofiber synthesis. The remodeling phase, the last stage of regeneration, involves the maturation of the regenerated myofibers and the reconstruction of the tissue.

Results from previous studies regarding the effectiveness of LIPUS in assisting muscle restoration are still inconclusive because some investigations demonstrated that no positive effect was found on regenerating skeletal myofibers with LIPUS treatment (Wilkin et al., 2004; Markert et al., 2005; McBrier et al., 2007). The different types of tissues, the various damage models, and the varying intensities and frequencies of LIPUS may be contributing factors to the opposed findings. Even though some research studies contested the value of LIPUS in the management of muscle damage, an increasing number of studies in recent years have elucidated its protective effects from different dimensions of muscle recovery.

Oxidative stress could apparently be increased in the first phase of the muscle healing process. The overproduction of reactive oxygen species is believed to be an important mechanism underlying muscle damage. Freitas et al. analyzed the effect of LIPUS on parameters of oxidative stress. Results showed that LIPUS decreased thiobarbituric acid-reactive substance (TBARS) levels and inhibited catalase and superoxide dismutase (SOD) activities on the first day after muscle contusion, which indicated that LIPUS protected the tissue from oxidative injury (Freitas et al., 2007). Meanwhile, LIPUS exhibited a stronger antioxidant impact when combined with other forms of therapy. In studies of Silveira et al. with two different injury models, the oxidative stress parameters, including superoxide anion, TBARS, protein carbonyls, superoxide dismutase, and catalase, were significantly decreased after associative treatment of LIPUS and dimethylsulfoxide (Silveira et al., 2010; Silveira et al., 2012). Martins et al. proved that LIPUS plus cryotherapy reduced oxidative stress in damaged muscle, leading to considerable tissue repair. More recently, limonene and diosmin were also demonstrated to be effective in reducing oxidative stress (Filho et al., 2018; Santos et al., 2020). Therefore, combining LIPUS with other chemical substances to expand its inherent therapeutic effect is a promising research direction. However, no existing study explores the underlying mechanism of how LIPUS mediates oxidative stress after acute injury. In addition to oxidative stress, an excessive inflammatory response to acute skeletal muscle injury can also be mediated by LIPUS. Alfredo et al. indicated that LIPUS could accelerate inflammation phases from the histomorphometric aspect (Alfredo et al., 2009). Results from Nagata et al. and Rennó et al. revealed that LIPUS promoted a downregulation of the inflammatory response, characterized by a decreased level of cyclo-oxygenase-2 (COX-2). On the contrary, Montali et al. discovered that LIPUS produced an upregulation of COX-2 after 7 and 13 days post-surgery in freeze-induced muscle injury (Montalti et al., 2013; Nagata et al., 2013). It can be explained by the fact that the effects of COX-2 vary at different stages of muscle recovery, while LUPUS suppressed the COX-2-mediated inflammatory response in the early stage and promoted COX-2-mediated muscle fiber regeneration in the later stage (Bolli et al., 2002). Signori et al. explained how LIPUS acted as an anti-inflammatory factor in the downregulation of inflammatory cells from the hematological dynamics aspect (Signori et al., 2011). In an *in vitro* experiment delivered by da Silva Junior et al., LIPUS was reported to be able to transfer the phenotype of macrophages from pro-inflammatory to anti-inflammatory, which could not only reverse the extensive inflammation after severe muscle injury but also attenuate tissue fibrosis (da Silva Junior et al., 2017). However, the underlying mechanism behind the phenotype conversion of RAW264.7 required further investigation. In a study regarding LIPUS-mediated anti-inflammatory effects on myocarditis by Zheng et al., caveolin-1 was significantly activated, and p38 mitogen-activated protein kinase (MAPK) and extracellular signal-regulated kinase (ERK) signaling were significantly suppressed, which demonstrated LIPUS treatment attenuated the aggressive inflammatory response in myocarditis by activating caveolin-1 and suppressing p38 MAPK/ERK signaling (Zheng et al., 2019).

The next stage is the repair phase, in which LIPUS stimulates the regeneration of muscle fibers. The data from Piedade et al., Chan et al., and Abrunhosa et al. uncovered that LIPUS promoted the formation of regenerative myofibers with the muscle laceration model *in vivo* and the C2C12 cell line *in vitro*. LIPUS-treated muscle presented an increase in the myogenic markers myogenin, desmin, actin proteins, and more arranged multinucleated myotubes (Piedade et al., 2008; Chan et al., 2010; Abrunhosa et al., 2014). Chongsatientam et al. proved that LIPUS hastened muscle recovery from angiogenesis aspects (Chongsatientam and Yimlamai, 2016). Nagata et al. reported that Pax7, a transcription factor specifically expressed in the nuclei of activating and proliferating satellite cells, was significantly upregulated by the intervention of LIPUS (Nagata et al., 2013). In a rat model of stress urinary incontinence (SUI), increased Pax7-positive cells observed by Yang et al. also indicated that LIPUS activated satellite cell myo-differentiation. They subsequently showed that the degree of p38 MAPK phosphorylation was elevated by LIPUS, revealing a new LIPUS-mediated pathway in satellite cells (Yang et al., 2019). P38 MAPK was reported to participate in activating dormant muscle satellite cells to contribute to adult myogenic differentiation (Brennan et al., 2021). It can be concluded that LIPUS could regulate different biological effects in different cells even through the same pathway, while p38 MAPK signaling was inhibited in RAW264.7 in the study of Zheng et al. Puts et al. disclosed another possible mechanism by which LIPUS promoted muscle regeneration. They discovered that LIPUS was able to modulate the mechanosensitive transcription factors AP-1 and Sp1 and the mechanosensitive protein YAP, leading to increased proliferation of C2C12 cells. In their successive study, they confirmed that these three transcription factors AP-1, Sp1, and TEAD were also activated in C2C12 mesenchymal precursor cells. Silencing of YAP expression reversed the therapeutic effects of ultrasound, further verifying this intracellular mechanism (Puts et al., 2016; Puts et al., 2018).

Although few studies have explored the effect of LIPUS on matrix remodeling in the final stage of recovery from skeletal muscle injury, some articles have proved that LIPUS can inhibit myocardial fibrosis in the rat model of myocardial injury, which has certain reference significance for the future study into LIPUS inhibiting fibrosis after skeletal muscle injury. Zhao et al. used LIPUS to stimulate Ang II-induced animal and cell culture models of cardiac hypertrophy and fibrosis. The results indicated that LIPUS significantly ameliorates left ventricular remodeling *in vivo* and cardiac fibrosis *in vitro* (Zhao et al., 2021a). At the same time, LIPUS could increase the expression of the mechanosensitive protein caveolin-1 and reduce the Ang II-induced release of inflammatory cytokines, while a caveolin-1 inhibitor blocked the LIPUS-induced downregulation of inflammation and the anti-fibrotic effects. According to their results, LIPUS could attenuate Ang II-induced myocardial fibrosis by reducing inflammation through a caveolin-1-dependent pathway. In their following study, LIPUS was applied to hypoxia-induced cardiac fibrosis *in vivo* and *in vitro*. They found that LIPUS dose-dependently attenuated hypoxia-induced cardiac fibroblast phenotypic conversion *in vitro* and ameliorated TAC-induced cardiac fibrosis *in vivo*. Meanwhile, LIPUS-induced anti-fibrotic impact and the downregulation of hypoxia-inducible factors were hindered by siRNA of the mechanosensitive protein TWIK-related arachidonic acid-activated K+ (TRAAK) channel. The fact that LIPUS can transform mechanical signals into intracellular biological signals not only through the conversion of membrane protein conformation but also through ion channels was brought to the surface by them (Zhao et al., 2021b). Nevertheless, a few mechanosensitive proteins have been well identified as being related to the stimulation of LIPUS.

**FIGURE 1:**
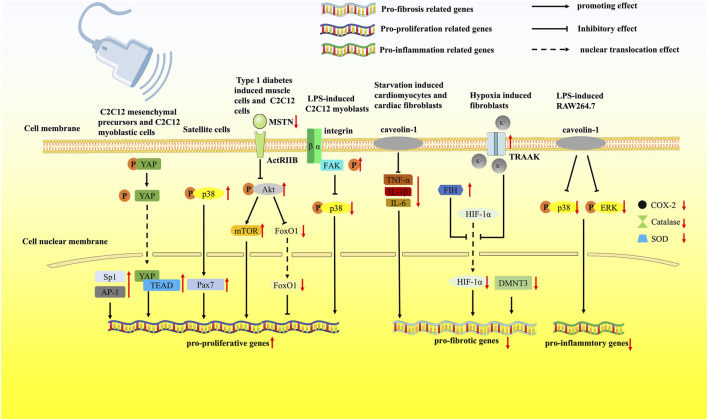
LIPUS regulates muscle regeneration *via* multiple pathways. LIPUS was reported to modulate the mechanosensitive transcription factors AP-1 and Sp1 and the mechanosensitive protein YAP, leading to increased proliferation of C2C12 cells. Pax7 was significantly upregulated by the intervention of LIPUS. Through the MSTN/Akt/mTOR and FoxO1 signaling pathways, LIPUS effectively ameliorated muscle atrophy. LIPUS activated the integrin/FAK signaling pathway and attenuated the phosphorylation of p38 MAPK, preventing LPS-induced muscle atrophy. LIPUS activated the TRAAK ion channel, which further inhibited the nuclear translocation of HIF-1α. LIPUS could increase the expression of the mechanosensitive protein caveolin-1 and reduce the Ang II-induced release of inflammatory cytokines. LIPUS treatment attenuated the aggressive inflammatory response by activating caveolin‐1 and suppressing the p38 MAPK/ERK signaling pathway.

In addition to muscle injury models, LIPUS was also widely researched in the treatment of other clinical muscle disease models. Through the evaluation of BrdU-positive satellite cells, Matsumoto et al. found that LIPUS inhibited the development of disuse muscle atrophy, which indicated that LIPUS exerted the anti-atrophy effects *via* the activation of satellite cells (Matsumoto et al., 2014). Tang et al. utilized rat type 1 diabetes as a muscle atrophy model and found that LIPUS effectively ameliorated muscle atrophy through the MSTN/Akt/mTOR and FoxO1 signaling pathways (Tang et al., 2017). Similarly, Sun et al. discovered that the MSTN/Akt/mTOR signaling pathway was the affected intracellular mechanism through which LIPUS improved unloaded-induced hindlimb muscle atrophy (Sun et al., 2021). Ueno et al. used LIPUS to treat LPS-induced atrophy of C2C12 cells and found that LIPUS activated the integrin/FAK signaling and attenuated the phosphorylation of p38 MAPK, thereby preventing LPS-induced muscle atrophy (Ueno et al., 2021). The results also reminded us of the fact that the biological effect delivered by LIPUS might depend on the status of the extracellular microenvironment, such as inflammation. The affected signaling pathways are listed in Figure 1; Table 1.

**TABLE 1:** Studies investigating the signaling pathways of LIPUS treatment for muscle regeneration.

Author	Study objective		Mechanism	Result
	Cell culture	Animal model		
Zheng et al. (2018)	LPS-induced RAW264.7	Mouse model of CVB3 induced myocarditis	LIPUS activated caveolin‐1 and suppressed p38 MAPK/ERK signaling	LIPUS treatment improved the survival rate, attenuated the ventricular dysfunction, and ameliorated the cardiac histopathological injury of CVB3‐infected mice. LIPUS treatment on RAW264.7 inhibited the expression of pro‐inflammatory cytokines
Yang et al. (2018)	✕	Rat model of stress urinary incontinence	LIPUS promoted myo-differentiation through activating the p38 MAPK signaling pathway	More robust striated muscle regeneration was observed in the LIPUS group. LIPUS activated the myo-differentiation of muscle satellite cells
Puts et al. (2015)	Murine C2C12 myoblastic cells	✕	LIPUS activated the mechanosensitive transcription factors AP-1, Sp1, and TEAD and the activated transcriptional coactivator YAP	Proliferation of the C2C12 cells was upregulated
Puts et al. (2016)	Murine C2C12 mesenchymal precursors	✕	LIPUS activated the mechanosensitive transcription factors AP-1, Sp1, and TEAD	LIPUS increased the expression of mechanosensitive genes. LIPUS was introduced for 5 min and upregulated the proliferation of C2C12 cells
Puts et al. (2018)	Murine C2C12 mesenchymal precursors	✕	LIPUS activated the mechanosensitive transcriptional coactivator YAP	LIPUS enhanced cell proliferation. YAP knockdown significantly reduced the cell growth induced by LIPUS
Zhao et al. (2021)	Starvation-induced neonatal rat cardiomyocytes and neonatal rat cardiac fibroblasts	Mouse model of Ang II-induced cardiac hypertrophy and fibrosis	LIPUS could ameliorate cardiac fibrosis *via* a caveolin-1-dependent pathway	LIPUS could ameliorate left ventricular remodeling *in vivo* and cardiac fibrosis *in vitro* by reducing Ang II-induced release of inflammatory cytokines
Zhao et al. (2021)	Hypoxia-induced neonatal rat cardiac fibroblasts	Mouse model of TAC cardiac fibrosis	LIPUS could prevent prolonged hypoxia-induced cardiac fibrosis through the TRAAK-mediated HIF-1α/DNMT3a pathway	LIPUS attenuated hypoxia-induced cardiac fibroblast phenotypic conversion *in vitro* and ameliorated TAC-induced cardiac fibrosis *in vivo*
Tang et al. (2017)	✕	Rat model of type 1 diabetes mellitus-induced gastrocnemius atrophy	LIPUS improved muscle atrophy induced by type 1 diabetes, and the MSTN/Akt/mTOR and FoxO1 pathways may play a role	LIPUS significantly improved type 1 diabetes-induced muscle atrophy, as evidenced by significantly enhanced muscle cross-sectional area, muscle mass, and strength
Sun et al. (2021)	Mouse myoblastic cell line and C2C12 cells	Rat model of unloading hindlimb gastrocnemius atrophy	LIPUS promoted protein synthesis through the MSTN/Akt/mTOR signaling pathway and stabilized alanine, aspartate, and glutamate metabolism	LIPUS promoted the proliferation and differentiation of myoblast C2C12 and prevented the decrease of the cross-sectional area of muscle fiber and gastrocnemius mass in hindlimb-unloading rats
Ueno et al. (2021)	LPS-induced murine skeletal muscle cells and C2C12 myoblasts	✕	LIPUS had preventive effects on inflammation-induced muscle atrophy through activating the phosphorylation of FAK and inhibiting the phosphorylation of p38 MAPK	LIPUS-attenuated myotube atrophy induced by LPS

CBV3, coxsackievirus B3; LPS, lipopolysaccharide; YAP, yes-associated protein; AP-1, activator protein-1; Sp1, specificity protein 1; TAC, transverse aortic constriction.

### Articular cartilage

Osteoarthritis (OA), characterized by progressive cartilage destruction and the development of arthralgia, stiffness, and restricted motion, is the most common disease in the middle-aged and senior populations (Glyn-Jones et al., 2015). An important manifestation in the molecular aspect of OA is the enhanced production of matrix-degrading enzyme matrix metalloproteinase-13 (MMP-13) and the reduced synthesis of aggrecan (ACAN) and collagen type II (Col II) (Zhu et al., 2019).

As an adjunctive therapy for this condition, LIPUS has been widely applied and researched. Although clinical trials have shown that LIPUS has a positive protective effect on the cartilage in arthritis, there is still controversy. For instance, the majority of clinical trials demonstrated increased joint symptoms, joint mobility, and medial tibia cartilage thickness and a reduction in inflammation in the LIPUS group (Loyola Sánchez et al., 2009; Yang et al., 2011; Jo et al., 2022), while controlled double-blind clinical studies from Ulus et al., Karakaş et al., and Cakir et al. revealed that LIPUS provided no additional benefits in knee pain, function, and femoral cartilage and synovial sac thickness in knee osteoarthritis (Ulus et al., 2012; Cakir et al., 2014; Karakaş et al., 2020). The clinical data from Yegin et al. showed that the improvement did not persist in the long term (Loyola-Sánchez et al., 2012). Therefore, further pilot trials are needed to explore optimal parameters for different degrees and stages of OA.

However, substantial pre-clinical animal and cell culture studies have confirmed the chondro-protective effects of LIPUS on cartilage (Korstjens et al., 2008; Naito et al., 2010; Uddin et al., 2016; Kamatsuki et al., 2019), including preventing degeneration, promoting matrix synthesis, and stimulating the migration, proliferation, and differentiation of chondrocyte precursor cells. Naito et al. have proved that LIPUS promotes the synthesis of the extracellular matrix such as COL II and ACAN in an OA rat model of anterior cruciate and medial collateral ligament transection and medial meniscus resection (Naito et al., 2010). Similarly, studies published by Kamatsuki et al. revealed that LIPUS exerted a compensatory impact on damaged meniscus by upregulating healing factors such as CCN2 in a rat model of meniscus defect, preventing meniscus from degenerative changes. Ji et al. found that LIPUS inhibited the expression of MMP-13 and promoted the expression of the inhibitor of metalloproteinases (TIMPs) in a rabbit OA model of an inner patellar ligament defect. In a recent study, Vahedai et al. found that LIPUS improved the histological appearance of cartilage and the expression of SOX9, COL II, and ACAN in a sheep model of cartilage defect (Vahedi et al., 2021). SOX9 is the pivotal transcription factor for COL II synthesis and chondrogenesis, which can not only maintain cell survival but also transcriptionally activate the genes for many cartilage-specific structural components (Lefebvre et al., 2019). Ding et al. demonstrated that through the upregulation of SOX9 expression, LIPUS promoted the synthesis and secretion of ECM and reduced cell apoptosis in human osteoarthritis (Ding et al., 2020). The aforementioned studies fully demonstrate the benign regulatory effects of LIPUS on matrix metabolism in different OA animal models.

Nashida et al. used LIPUS to treat cells of the human chondrosarcoma-derived chondrocytic cell line, and Sekino et al. (2018) used LIPUS to treat the mouse chondroprogenitor cell line ATDC5. Both demonstrated that LIPUS exerted a positive effect on chondrocyte stroma formation in a disease-free model. Therefore, LIPUS can modulate the matrix metabolism of cartilage not only *in vitro* and *in vivo* but also in disease and non-disease models. In addition to its effects on matrix production and degradation, certain studies have confirmed the role of LIPUS in promoting chondrocyte proliferation and differentiation (Sang et al., 2021). Above all, we can conclude that LIPUS has a positive effect throughout the whole process of cartilage regeneration.

Many studies have attempted to discover the underlying mechanisms behind LIPUS-promoting cartilage repair. In an experiment with the OA mouse model, Sang et al. explored that the FAK/p38 signaling pathways (activated) were strongly related to LIPUS intervention, and the inhibition of phosphorylated focal adhesion kinase (FAK) significantly reversed LIPUS-mediated cell proliferation, differentiation of chondrocytes, and LIPUS-mediated modulation of inflammation condition in OA mice (Sang et al., 2021). Nishida et al. used the human chondrocytic cell line (HCS)-2/8 and CCN2-deficient chondrocytes to treat with LIPUS. The data indicated that LIPUS exposure interacted with the membrane surface calcium channel TRPV4 to increase calcium influx in chondrocytes. The influx of calcium ions further promoted the phosphorylation of p38 mitogen-activated protein kinase (p38 MAPK) and extracellular signal-related kinase (ERK1/2), which ultimately promoted the increase of CCN2, a cartilage regeneration factor (Nishida et al., 2017).

However, the p38/ERK signaling pathway can also be suppressed by LIPUS treatment in OA pre-clinical experiments. Guan et al. treated primary chondrocytes isolated from the knee articular cartilage of 5-day-old mice with 20 min of treatment. The research revealed that LIPUS regulated the expression of vascular endothelium growth factor A (VEGFA), which was associated with cartilage degeneration, synovitis, and osteophyte formation. Further study with IL-1β-induced mouse primary chondrocytes demonstrated that this effect was achieved by suppressing the p38 MAPK signaling pathway (Guan et al., 2020). In addition, suppression of phosphorylation of ERK1/2 and p38 was also reported when the LIPUS was introduced to treat the rabbit knee OA model in the research of Li et al. (2011). Xia et al. found that after being treated with LIPUS in chondrocytes from OA rabbits, integrin β1 and FAK expressions were activated, and phosphorylated ERK 1/2 and phosphorylated p38 MAPK expressions were suppressed in the treatment group than in the OA group (Xia et al., 2015a). In addition, Cheng et al. demonstrated that LIPUS can affect the integrin-FAK-PI3K/Akt mechanochemical transduction pathway, leading to the alteration of ECM production in OA chondrocytes. The PI3K/Akt pathway is crucial to the pathologic development of OA and is intimately associated with chondrocyte matrix remodeling. In rabbit OA chondrocytes, activation of the PI3K/Akt pathway encouraged the production of ACAN and COL II. Thus, it can be concluded that through integrin, LIPUS inhibited the p38 MAPK signaling pathway and the ERK1/2 signaling pathway, finally promoting the PI3K/Akt signaling pathway (Cheng et al., 2014). Integrins are a family of cell surface stress receptors that mediate interactions between cells and the ECM (Sun et al., 2016). Another study from Xia et al. also proved that phosphorylated p38 was upregulated in normal chondrocytes but downregulated in OA chondrocytes after LIPUS stimulation (Xia et al., 2015b). Through the analysis of the aforementioned experimental data, it can be concluded that LIPUS exerts specific effects on the same pathway in different inflammatory situations or different cells.

Autophagy, as a degradation process that improves the intracellular environment, can also be affected by LIPUS and impact the migration of MSCs before chondrogenesis. The study by Wang et al. used bone marrow-derived mesenchymal stem cells (BMSCs) isolated from 8-week-old male Sprague–Dawley rats and found that LIPUS promoted the chondrogenic differentiation of BMSCs *via* inhibiting autophagy. Chondrogenesis was also promoted by the autophagy inhibitor 3-MA, indicating an inhibitory role for autophagy in the chondrogenic differentiation of MSCs (Wang et al., 2019). However, in OA mice injected with MSCs and treated with LIPUS, Xia et al. found that LIPUS activated autophagy and promoted MSC migration by augmenting the stromal cell-derived factor-1 (SDF-1)/CXC chemokine receptor 4 (CXCR4) signaling pathway. SDF-1 and CXCR4 have been demonstrated to be associated with the migration of MSCs during injury repair in many tissues. The introduction of autophagy blockers exhibited the opposite effects (Xia et al., 2021). As for LIPUS-optimizing chondrogenic progenitor cell migration, LIPUS stimulation was applied to treat OA-conditioned chondrogenic progenitor cells in the study of Jang et al. (2014). The results indicated that LIPUS promoted chondrogenic progenitor cell migration by activating the FAK pathway. Sekino et al. found that LIPUS induced collagen synthesis and the remodeling of ACAN *via* the activation of ERK1/2 in the mouse chondroprogenitor cell line ATDC5 (Sekino et al., 2018).

Stem cell therapy combined with LIPUS for soft tissue regeneration is a promising strategy. MSCs are highly proliferative, self-renewing cells, and their promising effects on cartilage regeneration have also been extensively studied (Richardson et al., 2016). Some insightful research discovered that with the stimulation induced by LIPUS, MSCs possessed a higher ability to regenerate and migrate to the injured area (Xia et al., 2022). Yamaguchi et al. first demonstrated the effectiveness of MSC injection combined with LIPUS irradiation compared with the treatment alone (Yamaguchi et al., 2016). In the study by Xia et al., the data first represented the fact that LIPUS promoted the chondrogenesis of TGF-β1-induced BMSCs through the integrin–mTOR signaling pathway *in vitro*. The result indicated that LIPUS promoted the chondrogenesis of TGF-β1-mediated BMSCs, and it was reversed following the addition of integrin inhibitors and the mechanistic target of rapamycin (mTOR inhibitors). Therefore, by promoting the mTOR signaling pathway, which played a key role in regulating chondrocyte proliferation and transformation (Zheng et al., 2014), LIPUS stirred MSCs’ chondrogenesis (Wang et al., 2019; Xia et al., 2021). Liao et al. also found that LIPUS could enhance the effects of BMSC-derived exosomes in IL-1β-induced chondrocytes by suppressing the NF-κB signaling pathway (Liao et al., 2021a). The research of Uddin et al. on the NF-κB pathway also revealed that LIPUS improved the condition of IL-1β-induced human chondrocytes by preventing the activation of the NF-κB signaling pathway, which further led to reduced expression of MMP-13 and metalloproteinase with thrombospondin motifs 5 (ADAMTS-5) in chondrocytes. IL-1β, as a pro-inflammatory cytokine, is strongly implicated in initiating and aggravating OA lesions. IL-1β also plays a significant role in the pathogenesis of OA and is substantially expressed in OA patients (Kapoor et al., 2011). ADAMTS-5 is a catabolic enzyme that promotes EMC degradation in cartilage (Xiong et al., 2021). In addition to the combination of LIPUS with stem cells, the combination of LIPUS with other therapies can also accelerate the recovery of cartilage damage, for example, the combination with fibroblast growth factor 2 in the study of Tang et al., with low-level laser therapy in the study of Paolillo et al. and with Prussian blue nanoparticles (Paolillo et al., 2018; Tang and Li, 2018; Zuo et al., 2021).

Recently, the molecular mechanism behind LIPUS-alleviating temporomandibular joint disorders (TMJDs) was also gradually uncovered. In one of two consecutive studies by Liang et al., LIPUS effectively inhibited chronic sleep deprivation-induced condylar cartilage injury in rats by encouraging chondrocyte regeneration and lowering the expression ratios of MMP-3/TIMP-1 and RANKL/OPG in condylar tissue. Consequently, LIPUS suppressed the degeneration of cartilage and osteoclast activity (Liang et al., 2019). In the following study, Liang et al. found that pathological changes in rat condylar cartilage tissue were significantly relieved when the LIPUS intervention was applied. In the LIPUS intervention group, PCNA-positive cells were significantly improved. PCNA is a well-known indication of the status of cell proliferation and is closely tied to DNA synthesis in cells (González-Magaña and Blanco, 2020). Moreover, He et al. proved that LIPUS protected TMJDs through upregulating ZNT-9 (Zn2+ exporters), which further downregulated the crucial ECM-degrading effector enzymes MMP-3, ADAMTS-5, and ADAMTS-8 (He et al., 2021). According to Yang et al., LIPUS therapy restored the functions of damaged mandibular chondrocytes both *in vitro* and *in vivo* through modulating the hypoxia-inducible factor (HIF) pathway (Yang et al., 2020).

**FIGURE 2:**
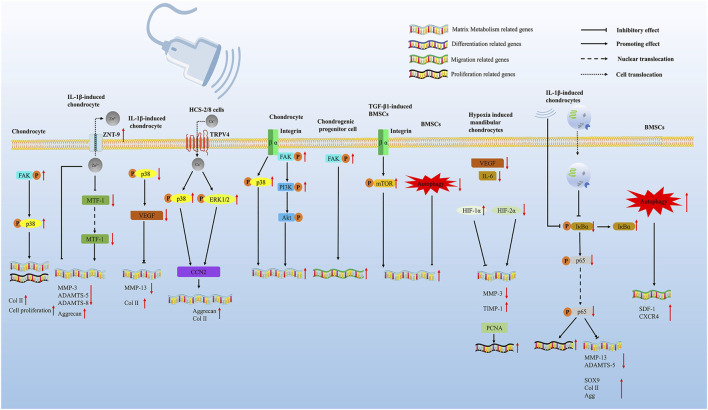
LIPUS regulates cartilage regeneration via multiple pathways. LIPUS promotes chondrocyte proliferation and differentiation by activating the FAK/p38 signaling pathway. LIPUS upregulates ZNT-9 (Zn2+ exporters), which further downregulates the crucial ECM-degrading effector enzymes MMP-3, ADAMTS-5, and ADAMTS-8 through inhibiting MTF-1. LIPUS regulated the expression of VEGFA *via* inhibiting the phosphorylation of p38 and further promoted cartilage degeneration. LIPUS exposure interacted with the membrane surface calcium channel TRPV4 to increase calcium influx in chondrocytes. The influx of calcium ions further promoted the phosphorylation of p38 MAPK and ERK1/2, ultimately promoting the increase of CCN2. LIPUS can affect the integrin-FAK-PI3K/Akt mechanochemical transduction pathway, leading to the alteration of ECM production in OA chondrocytes. LIPUS promoted chondrogenic progenitor cell migration by activating the FAK pathway. LIPUS promoted the chondrogenesis of TGF-β1-induced BMSCs through the integrin–mTOR signaling pathway. LIPUS promoted the chondrogenic differentiation of BMSCs by inhibiting autophagy. LIPUS enhances the effects of BMSC-derived exosomes on IL-1β-induced chondrocytes by suppressing the NF-κB signaling pathway. LIPUS therapy restored the functions of damaged mandibular chondrocytes both *in vitro* and *in vivo* through modulating the hypoxia-inducible factor (HIF) pathway. LIPUS activated autophagy and promoted MSC migration by augmenting the SDF-1/CXCR4 signaling pathway.

In the process of ultrasound treatment of cartilage injury, several intracellular signaling pathways are affected (Figure 2; Table 2), and then, protective biological effects are produced.

**TABLE 2:** Studies investigating the signaling pathways of LIPUS treatment for cartilage regeneration.

Author	Study objective		Mechanism	Result
	Cell culture	Animal model		
Sang et al. (2020)	Chondrocytes, including C28/I2 cells and CHON-001 cells	Mouse OA model of anterior cruciate ligament transaction	LIPUS alleviated OA and promoted chondrocyte proliferation and differentiation by activating FAK/p38 signaling	In OA mice, the arthritis score and weight-bearing abilities were improved by LIPUS. LIPUS declined the levels of inflammatory cytokines IL-6, IL-8, and TNF-α in synovial fluid of OA mice. LIPUS promoted chondrocyte proliferation and differentiation
Nishida et al. (2020)	Human chondrocytic cell line (HCS)-2/8 and CCN2-deficient HCS-2/8	✕	LIPUS increased Ca^2+^ influx by activating the TRPV4 channel. Increased Ca^2+^ further triggered p38 MAPK/ERK signaling, leading to increased production of CCN2	Gene expression of chondrocyte differentiation markers and CCN2 production were increased in cultured chondrocytes treated with LIPUS
Guan et al. (2020)	IL-1β-induced primary chondrocytes isolated from the knee articular cartilage of 5-day-old mice	Mouse OA model of destabilization of the medial meniscus	LIPUS reduced the expression of osteoarthritic chondrocyte-derived VEGFA through the suppression of p38 MAPK activity	LIPUS reduced the expression of VEGFA and catabolic genes *in vitro*. LIPUS decreased the expression of VEGFA and cartilage matrix loss and attenuated cartilage degeneration *in vivo*
Li et al. (2011)	✕	Rabbit OA model of ACLT	LIPUS promoted cartilage repair in OA through the downregulation of MMP-13 by suppressing ERK1/2 and p38 signaling	Early application of LIPUS could delay the degeneration of articular cartilage. MMP-13 expression was increased
Xia et al. (2015)	✕	Rabbit OA model of ACLT	LIPUS protected cartilage by activating integrin β1 and phosphorylated FAK and suppressing phosphorylated ERK 1/2 and phosphorylated p38 MAPK	Cartilage damage was less severe. The Mankin score was significantly lower. Early LIPUS treatment increased type II collagen expression but decreased MMP-13 expression
Cheng et al. (2021)	✕	Rabbit OA model of ACLT	LIPUS altered EMC production by activating the integrin-FAK-PI3K/Akt pathway in OA	LIPUS increased ECM-related genes but decreased MMP-1 and MMP-13 genes
Xia et al. (2015)	Chondrocytes isolated from femoral condyle of knee joints from OA and normal rabbits	Rabbit OA model of ACLT	LIPUS activated integrin β1 in normal and OA chondrocytes. Phosphorylated p38 was upregulated in normal chondrocytes but downregulated in OA chondrocytes	LIPUS increased type II collagen and decreased MMP-13 in normal and OA chondrocytes
Wang et al. (2017)	BMSCs isolated from 8-week-old male Sprague–Dawley rats	✕	LIPUS promotes MSC chondrogenesis by inhibiting autophagy	Higher number of type II collagen-positive cells was seen in the differentiating MSCs stimulated with LIPUS. Type II collagen, AGG, and SOX9 genes were also upregulated
Xia et al. (2021)	BMSCs isolated from 8-week-old male Sprague–Dawley rats	Rabbit OA model of ACLT	Migration of BMSCs was enhanced by LIPUS through the activation of autophagy	*In vitro* results suggested that LIPUS increased the expression of SDF-1 and CXCR4, and it promoted MSC migration. The *in vivo* results demonstrated that LIPUS enhanced the cartilage repair effects of BMSC therapy on OA
Jang et al. (2014)	Blunt-induced CPCs from tibial plateau of mature bovine stifle joints	✕	Beneficial effects of LIPUS on cartilage repair may be mediated by increased FAK activation in CPCs	LIPUS increased the number of CPCs into fibrin-filled defects
Sekino et al. (2018)	Mouse chondroprogenitor cell line ATDC5	✕	LIPUS induced matrix remodeling effect in ATDC5 cells through activating ERK1/2 signaling	LIPUS increased proteins and genes of EMC production-related factors, including Col II, Col X, and SOX9. Genes and protein levels of MMP-13 were reduced by LIPUS. Aggrecanase-5 was increased
Xia et al. (2017)	BMSCs obtained from 8-week-old male Sprague–Dawley rats and treated with TGF-β	✕	LIPUS promoted TGF-β1-induced chondrogenesis of BMSCs through the activation of the integrin-mTOR signaling pathway	Greater number of COL II-positive cells was observed. Protein expression of COL II, AGG, and SOX9 was increased
Liao et al. (2021)	IL-1β-induced primary chondrocytes harvested from the knee articular cartilage of 1–3-day-old Sprague–Dawley rats and incubated with BMSC-derived exosomes	Rat OA model of ACLT, partial medial meniscus resection, and intra-articular injected with BMSC-derived exosomes	Promotion of LIPUS on the effects of BMSC-derived exosomes on cartilage regeneration in OA could be related to the inhibition of IL-1β-induced activation of the phosphorylation of NFκB-p65 and IĸBα	LIPUS strengthened the promotion of BMSC-derived exosomes on OA cartilage regeneration *in vivo*. LIPUS strengthened the promoting effect of BMSC-derived exosomes on the proliferation of chondrocytes, cartilage matrix synthesis, and inflammation-inhibiting effect *in vitro*
Uddin et al. (2016)	IL-1β-induced C-28/I2 immortalized human chondrocytes	Cartilage explants harvested from patients receiving total knee joint replacement surgery	LIPUS suppressed IL-1β-induced activation of the phosphorylation of NFκB-p65 and IĸBα, leading to the reduced expression of MMP-13 and ADAMTS-5 in chondrocytes	LIPUS stimulation increased the proteoglycan content in human cartilage explants and inhibited IL-1β induced loss of proteoglycans *in vitro*. LIPUS stimulation increased the rates of chondrocyte migration and proliferation
He et al. (2020)	Chondrocytes isolated from the TMJDs of 3-week-old Wistar rats and treated with IL-1β	Rat TMJD-OA model of unilateral occlusal trauma	LIPUS protected the cartilage of TMJDs through the activation of ZNT-9	After LIPUS treatment, the cartilage showed a smoother surface and deeper ECM staining. RNA-Seq revealed that the expression of ADAMTS-8 was downregulated by LIPUS.
Yang et al. (2020)	Chondrocytes isolated from the mandibular condyle of 3-week-old male Wistar rats and treated with low oxygen tension	Rat TMJD-OA model by chronic sleep deprivation	HIF-1α expression was enhanced. HIF-2α was decreased	LIPUS reduced hypoxia-induced apoptosis in mandibular chondrocytes and promoted proliferation. LIPUS increased COL II and promoted chondrogenic capacity *in vitro*. LIPUS decreased IL-6/MMP-3 and increased TIMP-1

OA, osteoarthritis; Col II, type II collagen; FAK, focal adhesion kinase; CCN2, connective tissue growth factor; TRPV4, transient receptor potential vanilloid type 4; VEGFA, vascular endothelium growth factor A; MMP-13, matrix metalloproteinase 13; BMSCs, bone marrow-derived stem cells; AGG, aggrecan; SOX9, group-box gene 9; SDF-1, cell-derived factor-1; CXCR4, CXC chemokine receptor 4; CPCs, chondrogenic progenitor cells; EMC, extracellular matrix; ACLT, anterior cruciate ligament transection; ADAMTS-5, metalloproteinase with thrombospondin motifs 5; TMJD, temporomandibular joint disorder; ZNT, Zn^2+^exporters; TIMP-1, tissue inhibitor of metalloproteinase 1.

### Joint capsule

Joint stiffness can negatively impact the quality of life and everyday activities. Studies have revealed that atherogenic factors, especially those within the joint capsule, are crucial in the development of joint stiffness following immobilization (Itoi et al., 2016; Kim et al., 2021). Watanabe et al. histologically found that LIPUS irradiation improved the limitations on the range of motion in the rat model of immobilization by enlarging the gap between collagen fiber bundles of the posterior joint capsule (Watanabe et al., 2017). Itaya et al. proved the preventative effects of LIPUS on joint capsules in the rat model of an immobilized knee, including suppression of adhesion, elastic changes, fibrosis, inflammation, and hypoxia (Itaya et al., 2018).

As an integral part of the joint capsule, the pathological changes of the synovium in OA such as hyperplasia, inflammatory cell infiltration, increased angiogenesis, and fibrosis are closely related to joint condition and function (Mathiessen and Conaghan, 2017). Interventions are necessary to prevent disease progression and joint function decline. Different from the mini-invasive operation that delivers drugs *via* intra-articular injection, which possibly causes joint infection, LIPUS is safer and more convenient as a non-invasive method for synovial inflammation (Evans et al., 2014). The beneficial effects of LIPUS and the underlying mechanism on pathological changes of the synovium have been extensively reported. Nakamura et al. utilized the rat model of rheumatoid arthritis and IL-1β-induced rabbit knee synovial membrane cells to investigate the anti-inflammatory effects of LIPUS from multiple aspects (Nakamura et al., 2011). The results showed that LIPUS significantly downregulated cell proliferation *in vitro* and reduced COX-2-positive cells and synovial hyperplasia *in vivo*. Chung et al. found that LIPUS promoted neutrophil clearance by enhancing neutrophil extracellular trap (NET), a type of neutrophil death, and M2 macrophage phagocytosis, thereby suppressing synovial inflammation in the rat model of a complete Freund’s adjuvant-induced arthritis (Chung et al., 2016). According to Feltham et al.‘s research, LIPUS was able to control traumatic inflammation in rats with post-traumatic osteoarthritis by reducing the distribution and quantity of macrophages in the synovium and lowering the level of IL-1β in joint fluid (Feltham et al., 2021). However, the previous research failed to identify molecular mechanisms by which LIPUS acted as a protective factor for synovitis. Sato et al. revealed that LIPUS exposure participated in modulating cell apoptosis and survival of synovial membrane cells *via* the integrin/FAK/MAPK pathway (Sato et al., 2014). FAK is phosphorylated as a result of LIPUS-induced activation of mechanoreceptor integrin, which then downregulated ERK, JNK, and p38 phosphorylation, leading to an inhibitory effect on synovial cell proliferation. Zhang et al. focused their research on the molecular mechanism of LIPUS reducing IL-1β secretion. IL-1β was reported to be closely related to a pathological progression in synovitis. Their study indicated that LIPUS effectively ameliorated the gait patterns and synovial inflammation in a mouse model of destabilization of the medial meniscus, which was mainly related to the reduced production of IL-1β through enhancing sequestosome 1-dependent autophagy-mediated degeneration of pyruvate kinase 2 macrophages (Zhang et al., 2020). Liao et al. demonstrated that LIPUS directly inhibited TGF-β1-induced fibrotic response, cell proliferation of fibroblast-like synoviocytes, synovial fibrosis, and synovial hyperplasia of synoviocytes in the mouse model of destabilization of the medial meniscus. Further analysis indicated that LIPUS exerted anti-fibrosis effect through repressing the Wnt/β-catenin signaling pathway (Liao et al., 2021b).

**FIGURE 3:**
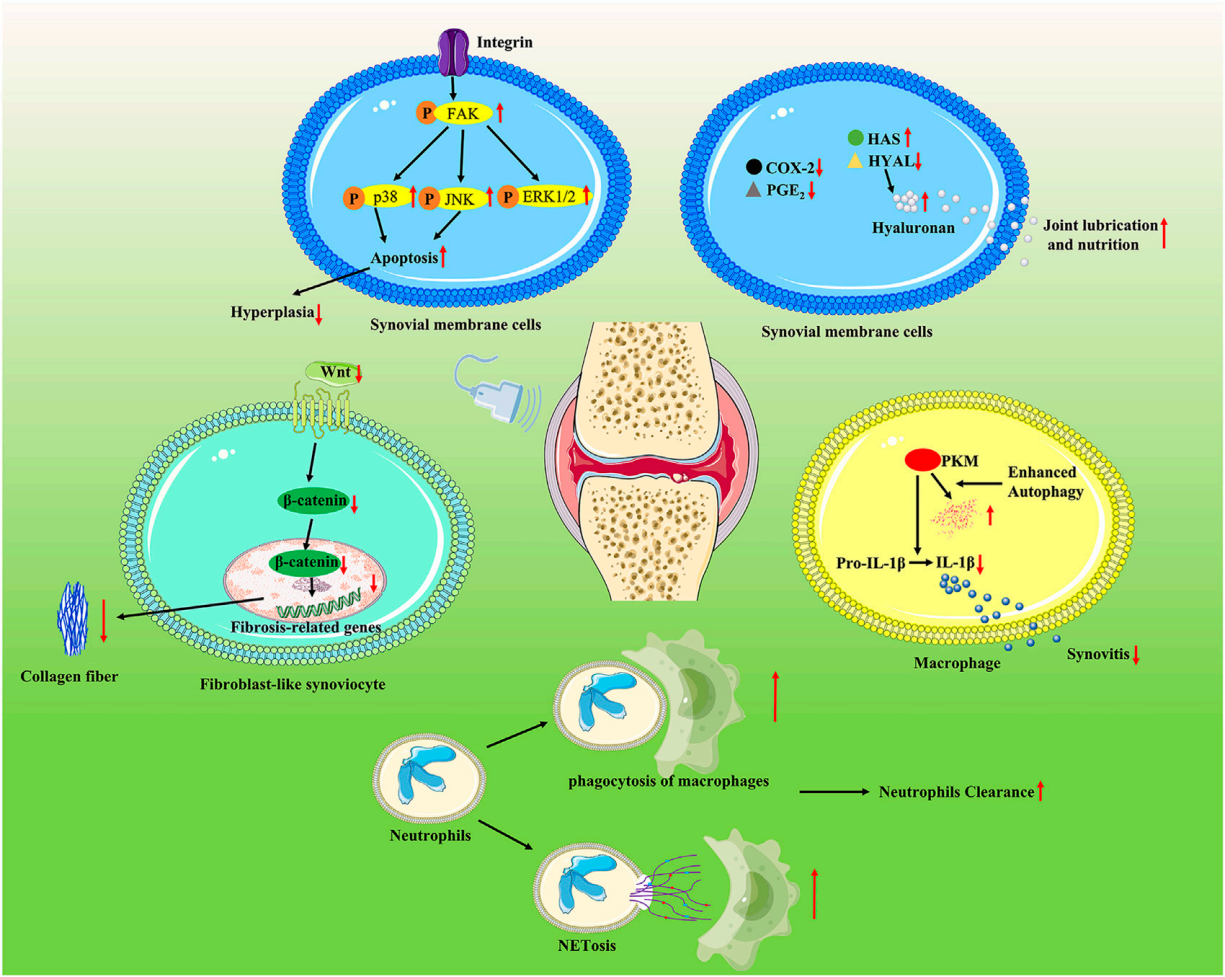
LIPUS regulates joint capsule conditions *via* multiple pathways. LIPUS exposure participated in modulating cell apoptosis and the survival of synovial membrane cells *via* the integrin/FAK/MAPK pathway. LIPUS increased the expression of hyaluronan synthase 2 and decreased the expression of hyaluronidase 2 while inhibiting the production of COX-2 and PGE2. LIPUS effectively ameliorated synovial inflammation by reducing the production of IL-1β through enhancing sequestosome 1-dependent autophagy-mediated degeneration of pyruvate kinase 2 macrophages. LIPUS promoted neutrophil clearance by enhancing NETosis and M2 macrophage phagocytosis and thereby suppressing synovial inflammation. LIPUS exerted an anti-fibrosis effect by repressing the Wnt/β-catenin signaling pathway.

The synovium is also responsible for the maintenance of lubricin and hyaluronic acid. Due to the lack of an inherent vascular or lymphatic supply, chondrocyte nutrition is mainly supported by the lubricin and hyaluronic acid produced by the synovium (Gupta et al., 2019). According to Nakamura et al., in IL-1β-stimulated synovial membrane cells, LIPUS increased the expression of hyaluronan synthase 2 and decreased the expression of hyaluronidase 2, while inhibiting the production of COX-2 and PGE2. Therefore, these results suggest that LIPUS enhanced the synthesis of hyaluronan, indicating an articular-protective response. The affected signaling pathways are listed in Figure 3; Table 3.

**TABLE 3:** Studies investigating the signaling pathways of LIPUS treatment for joint capsule conditions.

Author	Study objective		Mechanism	Result
	Cell culture	Animal model		
Chung et al. (2015)	Primary rat neutrophils	Rat synovitis model of intra-articular injection of a complete Freund’s adjuvant	LIPUS enhanced the NETs and resulted in neutrophil clearance by enhancing the phagocytosis of macrophages	LIPUS enhanced neutrophil clearance and macrophage activation
Sato et al. (2014)	Rabbit knee synovial membrane cell line HIG-82	✕	LIPUS exposure might be involved in cell apoptosis and the survival of synovial membrane cells *via* the integrin/FAK/MAPK pathway	✕
Zhang et al. (2019)	LPS-ATP-induced RAW 264.7 cells. Bone marrow cells from tibias and femurs of C57BL/6 mice	Mouse model of destabilization of the medial meniscus. Air pouch model	LIPUS inhibited the production of mature IL1B partially *via* SQSTM1-dependent autophagic degradation of PKM2 in LPS-ATP-treated macrophages	LIPUS ameliorated synovial inflammation and alleviated pain gait patterns *in vivo*. LIPUS inhibited the production of mature IL-1β *in vitro* and *in vivo*
Liao et al. (2021)	Mouse OA model of destabilization of the medial meniscus	TGF-β1 induced FLS isolated from synovial tissue of OA patients	LIPUS modulated OA-related synovial fibrosis, which is associated with its ability to repress the Wnt/β-catenin signaling pathway	Synovial fibrosis, synovial hyperplasia, and synoviocyte proliferation *in vivo* were decreased. TGF-β1-induced fibrotic response and proliferation of FLS were decreased

NTEs, neutrophil extracellular traps; PMNs, polymorphonuclear cells; and FLS, fibroblast-like synoviocytes.

## Discussion

**FIGURE 4:**
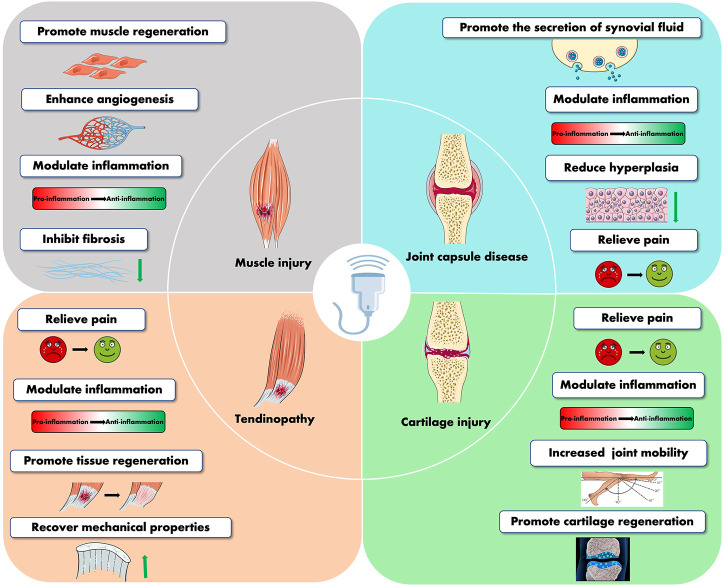
Summary of the therapeutic effects of LIPUS in musculoskeletal diseases.

It has been proven that LIPUS stimulation is an effective physical stimulus for musculoskeletal soft tissue injuries. It is less expensive and non-invasive than standard therapeutic ultrasonography or other physical agents, and it does not involve any pain or discomfort while being used. At present, LIPUS has been widely used in musculoskeletal injuries, and increasing beneficial feedback from patients has been obtained. Abundant data at the pre-clinical aspect supported the effectiveness of LIPUS as a modality that can modulate muscle regeneration after muscle injury, repair articular cartilage, protect joint capsules in osteoarthritic joints, and actively ameliorate tendon, ligament, and bone–tendon junction conditions (Figure 4). However, clinical trials have discovered that LIPUS cannot provide patients with the benefits we expected in this particular disease model. For example, several clinical trials failed to prove the effectiveness of LIPUS on ligament and muscle regeneration or on patients who suffer from tendinopathy and OA. The requirement for various treatment criteria for various diseases and the difficulties in effectively converting investigations at the animal and cellular levels to the clinic might be the root of this. In addition, a large variety of treatment parameters and treatment settings make it difficult to integrate and analyze all evidence-based research. Therefore, more tentative clinical trials are still needed to yield optimal therapeutic strategies for a specific stage of each musculoskeletal condition. In particular, for the application of LIPUS in the treatment of muscle injury, there are still no clinical trials to prove its effectiveness in muscle-lesioned patients. The future muscle injury clinical trial can be conducted by recruiting surgical patients who have recently undergone a surgical incision resulting in muscle injury. It is also important to consider how to increase the effectiveness of clinical transformation since clinical application is our ultimate objective. In addition, this review also has some shortcomings. First of all, we did not systematically summarize the therapeutic dosages of all trials and finally conclude the specific parameters that are reasonably applied to different musculoskeletal disorders. Second, in this review, we did not summarize the effects of LIPUS on angiogenesis and peripheral nerve regeneration since blood vessels and peripheral nerves also affect musculoskeletal soft tissue.

Despite several studies testifying to the efficacy of LIPUS, the theory on the mechanism of LIPUS in some disease models, such as tendons, ligaments, and joint capsules, is still superficial. Since the process of soft tissue repair is no more than inflammation, regeneration, and matrix remodeling stages, we may explore the mechanism of LIPUS by starting with the pathophysiology of soft tissue restoration. In addition, cell studies that simulate tendon, ligament, and joint capsule pathology are poorly designed. Therefore, the rational use of cell experiments is required to further verify the intracellular signaling pathway. From what we concluded, mechanically sensitive membrane surface receptors, such as integrin and caveolin, are key proteins in the conversion of mechanical energy signals into biological signals. In addition, the ion channels Piezo, TREK-1, TREK-2, TRAAK, and TRPV4 have been proven mechanically sensitive according to the research (Xu et al., 2020). If these ion channels are the mechanical–biological transduction target for LIPUS, further investigation is required. More mechanosensitive proteins need to be identified, whether they are related to the biological effects induced by LIPUS or not to reveal more potential mechanisms. These proteins can be ion channels, membrane surface receptors, and transcription factors. In addition, the intracellular phosphorylation of FAK, ERK, and p38 under LIPUS is the most studied intracellular factor at present.

By comparing different studies involving the same pathway, we found that LIPUS can exert different biological effects on the same signaling pathway in different cells and may also cause different biological effects in different extracellular microenvironments. For example, p38 MAPK phosphorylation was elevated by LIPUS in satellite cells, while p38 MAPK signaling was inhibited in RAW264.7. Therefore, we cannot rigidly define the effect of LIPUS on a particular signaling pathway. Specific microenvironmental conditions and cell-type conditions should be included to define LIPUS effects on the signaling pathway. However, based on the LIPUS-affected intracellular signaling pathways that have been revealed, we should also be adept at investigating the regulatory role of LIPUS on these signaling pathways in other biological settings and disease models. For example, many potential mechanisms have been discovered in the study of LIPUS on myocardial repair. If the LIPUS can exert the same biological effect in skeletal muscle repair, it requires further investigation.

Numerous types of research conducted in recent years have validated the beneficial effects of stem cell treatment on soft tissue healing (Qazi et al., 2019). However, cross-talk with the immune system, migration and survival of stem cells, and degree of differentiation of stem cells hamper the effectiveness of stem cell therapy on tissue regeneration (Balistreri et al., 2020). Therefore, combination therapy was highly recommended. If the combination therapy of LIPUS and stem cells could effectively promote the recovery of tissue damage, there still remains the following research: it is worthwhile to investigate if LIPUS and stem cells work better together to enhance soft tissue regeneration. It is also important to confirm if LIPUS is an effective adjunctive therapy to address these stem cell therapy-related issues.

## Perspective

There are several directions of research that deserve our attention. First, more clinical transformation trials are required to accelerate the clinical application of LIPUS, especially in muscle injury disease. Second, cell studies that simulate tendon, ligament, and joint capsule pathology are poorly designed. The intracellular signaling pathway needs to be further verified through the attentive application of cell studies. Next, to uncover further potential mechanisms for the biological impacts brought on by LIPUS, more mechanosensitive proteins must be found. Finally, it is worthwhile to investigate the combinative effect of LIPUS with other therapy strategies, such as stem cells, exosomes, and topical ointment. I believe that with the induction of LIPUS and its intrinsic therapeutic effect, the original effect of a certain therapeutic strategy will be augmented.
